# Effects of C-reactive protein rapid testing and communication skills training on antibiotic prescribing for acute cough. A cluster factorial randomised controlled trial

**DOI:** 10.1038/s41533-024-00368-9

**Published:** 2024-05-09

**Authors:** Carl Llor, Marta Trapero-Bertran, Antoni Sisó-Almirall, Ramon Monfà, Rosa Abellana, Ana García-Sangenís, Ana Moragas, Rosa Morros

**Affiliations:** 1 University Institute in Primary Care Research Jordi Gol (IDIAPJGol), Barcelona, Spain; 2 CIBER de Enfermedades Infecciosas. Instituto de Salud Carlos III, Madrid, Spain; 3 Research Unit for General Practice. Department of Public Health. University of Southern Denmark, Odense, Denmark; 4 Department of Economics and Business, Faculty of Law, Economics and Tourism, University of Lleida, Lleida, Spain; 5 Catalan Society of Family Medicine (CAMFiC). Fundació d’Atenció Primària, Barcelona, Spain; 6 Plataforma SCReN, UIC IDIAPJGol, Barcelona, Spain; 7 Universitat Autònoma de Barcelona, Bellaterra (Cerdanyola del Vallès), Spain; 8 Biostatistics, Department of Basic Clinical Practice, University of Barcelona, Barcelona, Spain; 9 University Rovira i Virgili, Reus, Spain; 10 Jaume I Health Centre, Institut Català de la Salut, Tarragona, Spain

**Keywords:** Clinical trial design, Outcomes research

## Abstract

This cluster randomised clinical trial carried out in 20 primary care centres in Barcelona was aimed at assessing the effect of a continuous intervention focused on C-reactive protein (CRP) rapid testing and training in enhanced communication skills (ECS) on antibiotic consumption for adults with acute cough due to lower respiratory tract infection (LRTI). The interventions consisted of general practitioners and nurses’ use of CRP point-of-care and training in ECS separately and combined, and usual care. The primary outcomes were antibiotic consumption and variation of the quality-adjusted life years during a 6-week follow-up. The difference in the overall antibiotic prescribing between the winter seasons before and after the intervention was calculated. The sample size calculated could not be reached due to the COVID-19 outbreak. A total of 233 patients were recruited. Compared to the usual care group (56.7%) antibiotic consumption among patients assigned to professionals in the ECS group was significantly lower (33.9%, adjusted odds ratio [aOR] 0.38, 95% CI 0.15–0.94, *p* = 0.037), whereas patients assigned to CRP consumed 43.8% of antibiotics (aOR 0.70, 95% CI 0.29–1.68, *p* = 0.429) and 38.4% in the combined intervention group (aOR 0.45, 95% CI, 0.17–1.21; *p* = 0.112). The overall antibiotic prescribing rates in the centres receiving training were lower after the intervention compared to those assigned to usual care, with significant reductions in β-lactam rates. Patient recovery was similar in all groups. Despite the limited power due to the low number of patients included, we observed that continuous training achieved reductions in antibiotic consumption.

## Introduction

Overuse of antibiotics has contributed to the development of antimicrobial resistance, with the highest burden being in low-resource settings^1^. It has been shown that most antibiotics are prescribed in primary care settings and that acute lower respiratory tract infections (LRTIs) represent one of the most common indications for their prescription^2^, despite the slight benefit obtained from their prescription^3^.

Different interventions have been evaluated in order to reduce the antibiotic consumption. An illness-focused approach seeks to emphasize a patient-centred management targeting at understanding the whole patient and sharing decisions, aligning decisions with patients’ wants, needs, and preferences rather than making an accurate diagnosis^4^. A disease-focused approach, on the other hand, seeks to improve diagnosis and the limited value of medical history and physical examination in differentiating between serious from self-limiting LRTIs, identifying those situations in which withholding antibiotic therapy can be safe^5,6^.

Catalan general practitioners typically diagnose LRTIs by assessing symptoms, signs, and clinical examination results, determining the need for antibiotic prescribing based on these findings. Although they may occasionally order a chest x-ray when pneumonia is strongly suspected, this practice is not consistently followed. The impact of the training in enhanced communication skills on reducing antibiotic prescribing for LRTIs has mainly been evaluated in several studies in low-prescribing countries^7,8^. The use of the C reactive protein (CRP) point-of-care testing has also been associated with a lower antibiotic prescribing. According to the latest Cochrane review, clinicians using this rapid test can safely reduce antibiotic prescribing for those LRTIs by 22%^9^. When this rapid testing is associated with a clinical guidance about how to interpret the CRP values this reduction is greater. Limited evidence exists regarding the effectiveness of these various approaches in nations with elevated antibiotic prescription rates. While CRP testing is widely used in most European countries for the management of LRTIs, they are not yet routinely used in southern Europe^10^. Similarly, clinicians, once they are well established in clinical practice, rarely receive specific training to enhance their communication skills^11^. We thereby evaluated the effect of a continuous training in enhanced communication skills plus interactive booklet for patients and professionals’ use of CRP test, separately and combined, on antibiotic consumption for patients with LRTI and on patient recovery.

## Results

**Fig. 1:**
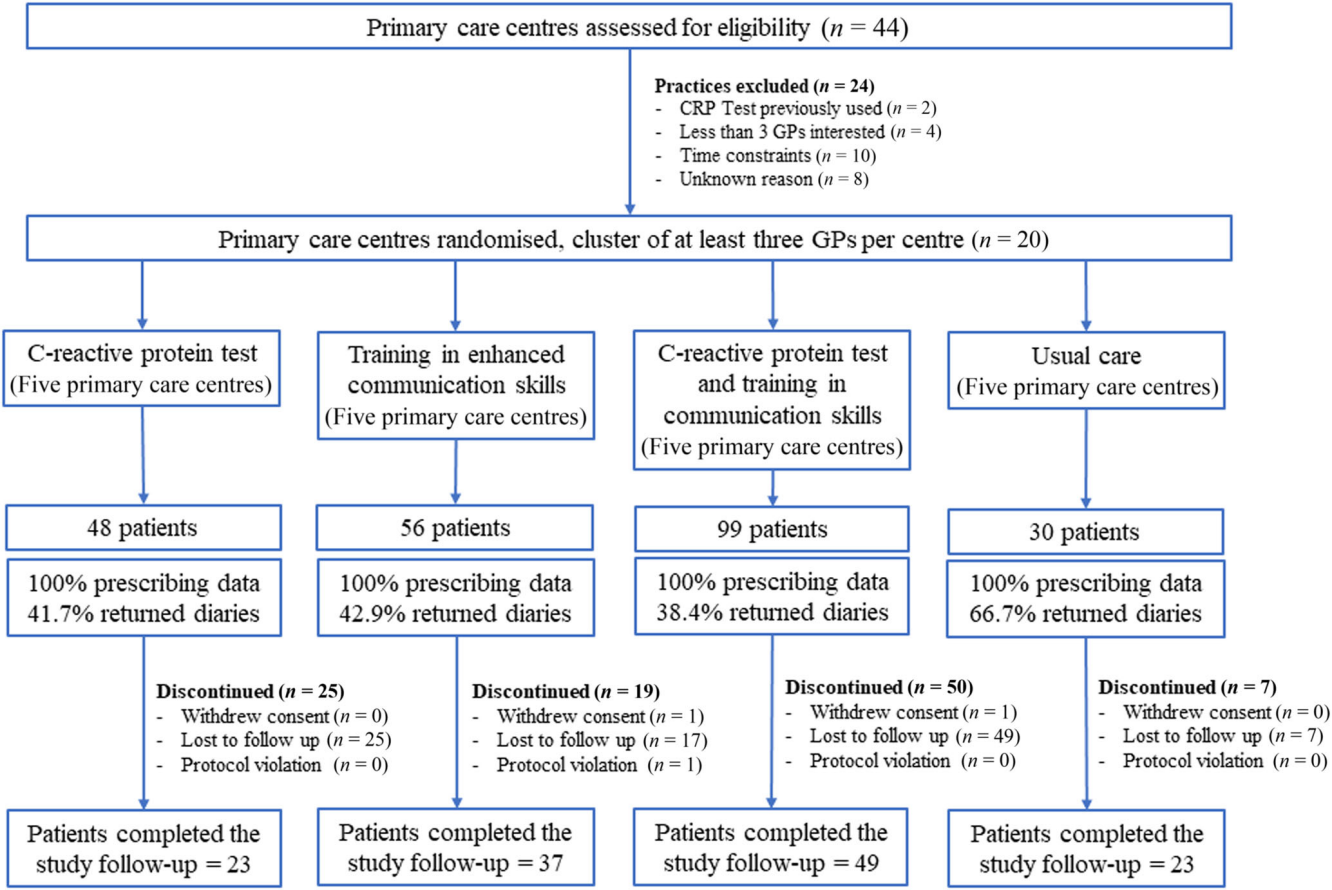
Patient randomisation flowchart showing reasons for exclusion from randomisation, patients recruited per arm, and follow-up. CRP C-reactive protein, GP general practitioner.

Of 44 primary health centres approached, 20 agreed to participate (Fig. 1). The characteristics of the centres were similar across the groups. A total of 231 professionals participated in this project, with 181 undergoing the allocated intervention and 50 were assigned to the usual care arm (Supplementary Table 1). The recruitment of patients in the different participating centres stopped on March 13, 2020, following the onset of the COVID-19 pandemic. As in many other countries, the Catalan healthcare system experienced major disruptions caused by the COVID-19 pandemic resulting in a significant change in the organisation of the primary healthcare centres and face to face visits were severely restricted. Despite efforts to substantially simplify the trial before the winter season in 2021, the subsequent waves of mild cases of COVID-19 infections continued disrupting the normal function of the primary care practices and did not allow resuming patient recruitment. As the funding body of the project had a time restriction in completing the study, the research team decided to stop the clinical trial in April 2022.

The expected sample size was not met. In total, 233 patients with LRTI were recruited, and data for the primary outcome measure were available in 231 patients. Symptom diary data were available for 102 patients (43.8% of participants). Table 1 shows the patients’ baseline characteristics. Patients assigned to the double intervention were older and more men were recruited compared to the other groups. The presence of comorbidities was similar across the different groups. More complete information on the baseline symptoms and signs of the patients participating in the trial is shown in Supplementary Table 2.

**Table 1:** Baseline characteristics of patients assigned to practices allocated to point of care testing for C-reactive protein, training in enhanced communication skills, combined interventions, and usual care.

	All (*n* = 233)	Communication skills training (*n* = 56)	CRP training (*n* = 48)	CRP + communication skills training (*n* = 99)	Usual care (*n* = 30)
Male gender*	83 (35.9)	23 (41.1)	9 (19.1)	43 (43.9)	8 (26.7)
Age*, mean (SD), in years	58.7 (17.9)	57.2 (19.8)	52.9 (16.5)	62.3 (17.6)	58.5 (14.7)
Active smoker	50 (21.7)	13 (23.2)	9 (19.1)	17 (17.6)	11 (36.7)
Comorbidities:					
- Lung disease (COPD or asthma)	55 (26.2)	12 (21.4)	13 (27.1)	27 (27.2)	9 (30.0)
- Heart disease	70 (30.0)	18 (32.1)	11 (22.9)	32 (32.3)	9 (30.0)
- Diabetes	24 (10.3)	5 (5.4)	9 (18.8)	11 (11.1)	1 (3.3)
Cough, median (IQR)	4.0 (3.0–5.0)	4.0 (3.8–5.3)	5.0 (4.0–5.0)	3.0 (3.0–5.0)	4.0 (3.0–5.0)
Breathlessness, median (IQR)	2.0 (0.0–4.0)	3.0 (0.0–5.5)	2.0 (0.0–4.3)	2.0 (0.0–3.0)	4.0 (1.8–4.3)
More than one respiratory tract infection in the last year	55 (24.3)	15 (27.3)	13 (29.5)	21 (21.6)	6 (20.0)
Hospitalisation in the last year due to respiratory diseases	6 (2.6)	1 (1.8)	2 (4.4)	2 (2.0)	1 (3.3)
Vaccination against flu in the last year	129 (56.3)	32 (57.1)	31 (67.4)	51 (52.6)	15 (50.0)
Pneumococcal vaccination in the last five years	55 (24.4)	13 (23.2)	6 (13.3)	26 (27.7)	10 (33.3)
Sick leave given	41 (17.6)	4 (7.1)	12 (25.0)	20 (20.2)	5 (16.7)
Diagnosis:					
- Acute bronchitis	165 (70.8)	35 (62.5)	38 (79.2)	70 (70.7)	22 (73.3)
- Pneumonia	13 (5.6)	2 (3.6)	3 (6.2)	7 (7.1)	1 (3.3)
- COPD exacerbation	20 (8.6)	4 (7.1)	7 (7.1)	5 (10.4)	4 (13.3)
- Other diagnosis	35 (15.1)	15 (26.8)	2 (4.2)	15 (15.1)	3 (10.0)
CRP rapid tests performed^‡^	103 (70.1)	0 (0.0)	44 (91.7)	59 (59.6)	0 (0.0)
Informative leaflets discussed and given to the patient^‡^	125 (80.6)	36 (64.3)	0 (0.0)	89 (89.9)	0 (0.0)
Antibiotic prescribing:					
- Immediate prescribing*	76 (39.9)	14 (25.5)	14 (29.2)	33 (33.7)	15 (50.0)
- Delayed prescribing	7 (3.0)	0 (0.0)	0 (0.0)	7 (7.1)	0 (0.0)
Non-antibiotic therapy:					
- Paracetamol or NSAIDs	71 (30.5)	15 (26.8)	14 (29.2)	35 (35.4)	7 (23.3)
- Antitussives or mucolytics	62 (26.6)	19 (33.9)	12 (25.0)	23 (23.2)	8 (26.7)
- Inhaled β2-agonists or anticholinergics	89 (38.2)	31 (37.5)	25 (52.1)	30 (30.3)	13 (43.3)
- Inhaled corticosteroids	48 (20.6)	14 (25.0)	13 (27.1)	14 (14.1)	7 (23.3)
- Natural remedies	37 (15.9)	9 (16.1)	3 (6.3)	21 (21.3)	4 (13.3)
- Other therapies	12 (5.2)	3 (5.3)	4 (8.3)	5 (5.0)	0 (0.0)

Values are in *n* (%) unless stated otherwise.

*COPD* chronic obstructive pulmonary disease, *CRP* C-reactive protein, *IQR* interquartile range, *NSAID* non-steroidal anti-inflammatory drug, *SD* standard deviation.

^*^p < 0.05; ^‡^*p* < 0.001.

The interactive leaflet was handed out and discussed with 125 patients (64.3% in the group assigned to enhanced communication skills alone and 89.9% in the combined strategy). The CRP was measured in 103 patients recruited by healthcare professions allocated to point of care testing (91.7% of the patients in the combined strategy group and 59.6% of those allocated to CRP alone). Overall, 65.3% of patients had test results of <20 mg/l, 13.9% of 20–40 mg/l, 13.9% of 41–100 mg/l and 7% of >100 mg/l. Standard laboratory testing for CRP was not ordered for any patient in the control group or in those allocated to enhanced communication skills.

### Primary outcomes

Within the first 6 weeks after the index consultation 56.7% of the patients visiting professionals assigned to usual care consumed antibiotics. Compared to the usual care group, antibiotic consumption among patients assigned to healthcare providers in the enhanced communication skills was significantly lower (33.9%, adjusted odds ratio [aOR] 0.38, 95% confidence interval [CI] 0.15–0.94, *p* = 0.037), whereas patients assigned to professionals in the CRP training group consumed 43.8% of antibiotics (aOR 0.70, 95% CI 0.29–1.68, *p* = 0.429) and those with the combined intervention had a consumption rate of 38.4% (aOR 0.45, 95% CI, 0.17–1.21; *p* = 0.112) (Table 2). The mean difference in the EQ-5D-5L questionnaire score in the first two weeks was similar in the four arms. Compared to the no intervention group, the adjusted mean difference was −0.01 in the communication enhancement group (95% CI, −0.07–0.05), 0.05 (95% CI, −0.02–0.12) in the CRP group and −0.03 in the combined intervention group (95% CI, −0.07–0.02). No statistically significant differences were observed in the differences in the visual analogue scale scores of the EQ-5D-5L questionnaire across the four trial arms (Table 3). The secondary outcome results are described in the Supplementary Material and Supplementary Tables 3 and 4.

**Table 2:** Effectiveness of C-reactive protein and enhanced-communication training in reducing antibiotic consumption rates withing the first six weeks, antibiotic prescribing at the index consultation and antibiotic appropriateness based on antibiotic prescribing at the baseline consultation.

Total (*n* = 233)^a^	Usual care (*n* = 30)	Communication skills training (*n* = 55)	CRP training (*n* = 48)	Communication skills and CRP training (*n* = 98)
Primary outcome: antibiotic consumption during the 6-week follow-up				
Crude percentage	56.7% (17/30)	33.9% (19/56)	43.8% (21/48)	38.4% (38/99)
OR (95% CI)	1.00	0.40 (0.16–0.99, *p* = 0.047)	0.60 (0.23–1.51; *p* = 0.28)	0.48 (0.21–1.10; *p* = 0.08)
Adjusted OR (95% CI)^b^	1.00	0.38 (0.15–0.94, *p* = 0.037)	0.70 (0.29–1.68, *p* = 0.43)	0.45 (0.17–1.21; *p* = 0.11)
Secondary outcomes				
Antibiotic prescribing at the index consultation				
Crude percentage	50.0% (15/30)	25.5% (14/55)	29.2% (14/48)	33.7% (33/98)
OR (95% CI)	1.00	0.34 (0.13–0.87, *p* = 0.024)	0.41 (0.16–1.06; *p* = 0.07)	0.51 (0.22–1.17; *p* = 0.11)
Adjusted OR (95% CI)^b^	1.00	0.36 (0.14–0.92, *p* = 0.033)	0.49 (0.19–1.23; *p* = 0.13)	0.51 (0.19–1.23, *p* = 0.20)
Antibiotic appropriateness based on antibiotic prescribing at the index consultation				
Crude percentage	63.3% (19/30)	83.6% (46/55)	72.9% (35/48)	76.5% (75/98)
OR (95% CI)	1.00	2.91 (1.03–8.47, *p* = 0.044)	1.55 (0.57–4.19, *p* = 0.39)	1.88 (0.76–4.54, *p* = 0.17)
Adjusted OR (95% CI)^b^	1.00	2.56 (0.80–8.25, *p* = 0.11)	1.40 (0.36–5.42, *p* = 0.32)	1.65 (0.54–5.05, *p* = 0.38)

*CI* confidence interval, *CRP* C-reactive protein, *OR* odds ratio.

^a^Missing data for two participants (1 in the communication skills group and 1 in the combined intervention group).

^b^The adjusted model additionally controlled for age, sex, smoking, major cardiovascular or respiratory comorbidity, diabetes, and clustering by practice.

**Table 3:** Effectiveness of C-reactive protein and enhanced-communication skills training in the variation of the EQ-5D-5L questionnaire and visual analogue scale scores in the first two weeks.

Total (*n* = 178)	Usual care (*n* = 25)	Communication skill training (*n* = 40)	CRP training (*n* = 35)	Communication skill and CRP training (*n* = 78)
*Variation in the EQ-5D-5L questionnaire score in the first two weeks*				
Variation, mean (SD)	0.06 (0.11)	0.06 (0.17)	0.11 (0.15)	0.03 (0.13)
Mean difference (95% CI)	1.00	−0.01 (−0.08–0.07, *p* = 0.88)	0.05 (−0.02–0.13; *p* = 0.18)	−0.03 (−0.1–0.03; *p* = 0.34)
Adjusted mean difference (95% CI)^a^	1.00	−0.01 (−0.07–0.05, *p* = 0.76)	0.05 (−0.02–0.12; *p* = 0.18)	−0.03 (−0.07–0.02; *p* = 0.27)
*Variation in the visual analogue scale score in the first two weeks*				
Variation, mean (SD)	12.6 (16.6)	17.0 (19.3)	18.8 (22.3)	13.7 (20.9)
Mean difference (95% CI)	1.00	4.40 (−5.80–14.60, *p* = 0.40)	6.16 (−4.45–16.76; *p* = 0.25)	1.07 (−8.09–10.24; *p* = 0.82)
Adjusted mean difference (95% CI)^a^	1.00	3.22 (−4.49–10.94, *p* = 0.41)	3.94 (−7.19–15.07; *p* = 0.49)	0.29 (−6.94–7.52; *p* = 0.94)

*CI* confidence interval, *CRP* C-reactive protein, *SD* standard deviation, *VAS* visual analogue scale.

^a^The adjusted model additionally controlled for age, sex, smoking, major cardiovascular or respiratory comorbidity, diabetes, and clustering by practice.

### Differences in overall antibiotic consumption

**Fig. 2:**
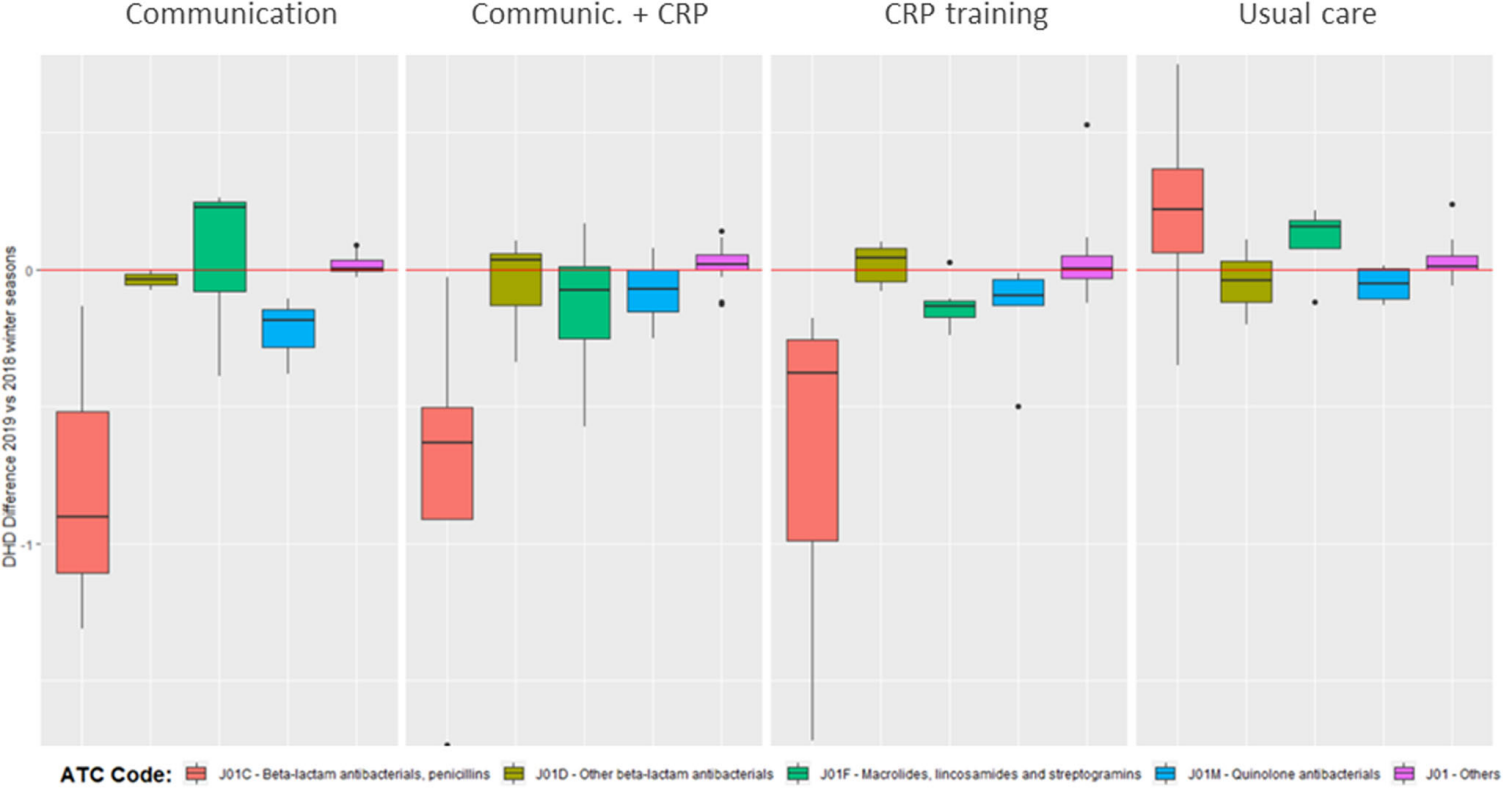
Variation of the daily defined doses per 1000 inhabitants and day of the J01 group antibiotics prescribed in the participating practices between the 2019–2020 and 2018–2019 winter seasons.

The overall prescribing rate of antibiotics corresponding to β-lactams (J01C) was significantly lower in the three groups which were intervened whereas a slight increase was observed among the primary care centres assigned to usual care (Fig. 2). The greatest reduction in the prescribing of β-lactams was observed in the centres assigned to the communication skills, followed by those in the combined group and those assigned to CRP testing, with these results being aligned with the results of the primary outcome.

## Discussion

The excessive use of antibiotics for the treatment of self-limited LRTIs is an important health issue. In this context the development and implementation of strategies to reduce unnecessary antibiotic treatment and improve antibiotic appropriateness are of paramount importance. Our results show a trend indicating that patients assigned to doctors who undergo continuous healthcare professional training in enhanced communication skills, as well as the utilisation of CRP point-of-care testing, consumed fewer antibiotics, without adversely affecting recovery or satisfaction. However, this effect was only statistically significant in the group exposed to communication skill enhancement training, mainly because of the limited number of patients recruited. Moreover, this reduction in antibiotic consumption and prescribing in the three intervention arms was further supported by the manifest reduction in the overall prescribing rates of mainly β-lactams in the 15 primary health centres assigned to any intervention compared to the rates of the previous year, whereas this overall reduction was not observed in centres assigned to usual care.

The major limitation of this study was its limited power due to the low number of patients included. This can lead to false negative results because of the width of the confidence intervals. The further recruitment of cases was unfeasible because of the reorganisation of primary care healthcare, oriented to the prioritisation of care for COVID-19 patients, the fact that cases of cough due to LRTIs clearly overlapped with COVID-19 infection, and because of the professionals’ pandemic fatigue^12^. All the cases were included at the beginning of the recruitment period, and we cannot ensure that the results would have been the same if we had been able to continue the trial. Some of the baseline characteristics were significantly different across groups due to the low number of patients included. However, we cannot assume that the severity of the patients differed across the groups.

Previous studies have examined antibiotic consumption within the first 28 days after the index visit. In contrast, our trial extended the timeframe to 6 weeks after the index consultation. While this extension allowed us to include patients still experiencing symptoms after four weeks, it also presents a limitation by reducing comparability with other studies. Another important limitation was the low number of diaries returned as less than half were recovered, mainly due to loss when the pandemic broke out. However, this did not affect the main outcome of the trial and only some of the secondary outcomes were based on self-reported information contained in these symptom diaries. Despite all these adversities, we were able to generate results that are certainly important for the future of the management of these infections in primary care.

We cannot discard a selection bias when clinicians recruited patients with infectious LRTI. A cluster design was used to maintain contamination within centres to a minimum and individual randomisation was not an option for our study design because our interventions were targeted at the level of healthcare professionals, and once trained in any intervention, professionals could not be expected to switch at random between any of these interventions and usual consulting practice^13,14^. However, the pragmatic nature of our clinical trial enhances the generalisability of the results. Our study included the full range of patients with LRTIs seen by healthcare professionals, with all the associated diagnostic uncertainty. Despite these limitations, we observed a reduction in the percentage of antibiotics consumed among patients assigned to the intervention groups. This finding is supported by the variation in the number of antibiotics prescribed in the intervened centres before and after the study, mirroring the results of the clinical trial, assuming that even though the necessary number of patients was not recruited, the professionals at the centre had received training in CRP and/or communication skills, which they could apply to their routine clinical practice to adjust antibiotic prescription.

Two main randomised clinical trials with the same aim have been published so far, one in the Netherlands and the other one, the GRACE-INTRO study, in different European nodes^7,8^. However, the effect of the communication skills training was greater in the former study, as the intervention was more intense and continuous and was not only limited to a single online training lasting approximately one hour before the trial started. The STAR study, which included five stages of web-based training in advanced communication skills, resulted in a 4% decrease in antibiotic prescribing in Welsh practices over a year^15^. Our study also achieved significant reductions among professionals exposed to enhanced communication skills plus use and discussion of interactive leaflets for patients. These results indicate that more intensive training yields more effective antibiotic use reduction.

A 3.5-year follow-up analysis of the Dutch study showed that only doctors assigned to enhanced communication skills continued presenting significantly lower antibiotic rates, compared to those assigned to the point of care test^16^. At the 12-month follow-up of the GRACE-INTRO study, only doctors assigned to the enhanced communication skills group remained efficacious for reducing prescribing for LRTIs^17^. One common finding in both studies is that doctors exposed to CRP used the test much less in the long term. Our study showed significant reductions in the group of professionals trained in communication skills, but not those using CRP rapid testing, similar to the results observed in the previous follow-up studies.

Considerable evidence shows that non-clinical factors, like patient expectations, time constraints, and relationship maintenance, influence antibiotic prescription decisions^18^. Likewise, diagnostic uncertainty leads to higher antibiotic prescribing rates for non-recommended conditions like LRTIs^19^. Training healthcare providers in communication skills, which involve eliciting patient concerns and offering evidence-based information, can boost patient confidence in self-management and when to reconsult.

While our assessment focused on patients with LRTIs, the interventions explored in this study may have broader applications, extending beyond LRTI cases. Both approaches could be used for most common infectious conditions in primary care. This is also supported by the by the notable decrease in antibiotic prescribing rates for J01C antibiotics, particularly β-lactams, which comprised a significant portion of antibiotics prescribed for LRTIs across all participating centres, regardless of the assigned intervention.

In conclusion, despite the limited number of cases recruited as a consequence of the COVID-19 outbreak, we observed a trend towards reduced antibiotic consumption among patients assigned to the intervention groups, but the effect was statistically significant only in the group allocated to enhanced communication skills with the potential use of interactive informative leaflets. Prescribing fewer antibiotics in the intervention groups did not result in poorer patient outcomes. These effects could also have major implications for the management of LRTI in countries with high antibiotic prescribing rates.

## Methods

### Study design

This study was a cluster randomised, factorial, controlled trial carried out in the city of Barcelona, Catalonia (NCT registry, ID: NCT03931577).

### Study participants

Patients who fulfilled the inclusion criteria were given written and verbal information about the study and were asked to provide written informed consent. Patients older than 18 years with a first consultation for acute cough (new cough or worsening of a previous cough) of up to 3 weeks’ duration as the predominant symptom, which the clinician believed to be an infectious LRTI, were recruited. Patients with a working diagnosis of a non-infective disorder, such as heart failure, pulmonary embolus, or oesophageal reflux, use of antibiotics in the previous two weeks, hospitalisation due to an acute LRTI, immunological deficiencies, inability to provide informed consent and/or unable to follow the study procedures were all excluded. Pneumonia and acute exacerbations of chronic obstructive pulmonary disease (COPD) were not deemed as exclusion criteria, as they were included in the definition of LRTI. The four groups allocated were (a) healthcare professional training in enhanced communication skills, (b) training in the use of a point-of-care CRP test testing, (c) combined training in CRP testing and enhanced communication skills, and (d) usual care. Central ethical approval has been obtained from the Research Ethics Committee IDIAP Jordi Gol (reference approval no. P18/227).

### Randomisation

Eligible primary care centres were randomised into the four groups of 5 practices per arm, stratified by two variables: (a) socioeconomic level based on the 2015 socio-demographic index issued by the Catalan government^20^, and (b) baseline daily defined dose of the systemic antibiotic prescribing rate of the different participating primary care centres (J01 therapeutic subgroup of the Anatomical Therapeutic Chemical Classification System) corresponding to the year 2018. Randomisation of primary care centres was achieved by computer generation of random numbers. A total of 20 primary care health centres of the Barcelona region were included and at least 3 general practitioners and 3 nurses participated in each practice. Every director from each practice provided their approval to take part in the study before starting it.

### Outcomes

The primary outcome measure was double: (a) antibiotic consumption within the first six weeks, as documented in the case report form and double-checked by on the electronic medical history, and (b) the variation in quality-adjusted life years, which was collected by means of the EQ-5D-5L questionnaire and the visual analogue scale within the two first weeks. Although most studies consider a timeframe of four weeks for the first co-primary variable, we decided to extend this time to up to six weeks as 21% of patients with LRTI still have cough after this period^21^. Moreover, we investigated the difference in the overall antibiotic prescribing rates (anatomical therapeutic chemical classification J01) in the 20 centres participating in the winter season when the clinical trial was performed (November 2019 to February 2020) compared to the same period one year earlier. We differentiated the common antibiotic families most prescribed for LRTIs: J01C (β-lactams), J01D (cephalosporins), J01F (macrolides) and J01M (quinolones). Secondary outcomes are described in the Supplementary Material.

### Data collection

Study data were collected and managed using REDCap (Research Electronic Data Capture) electronic data capture tools hosted at *Fundació Institut Universitari per a la recerca a l’Atenció Primària de Salut Jordi Gol i Gurina* (IDIAPJGol). REDCap is a secure, web-based software for research data capture, offering intuitive interfaces for data entry, audit trails for tracking, automated export, and data integration^22,23^. Only site investigator teams had data access and editing rights.

### Interventions

The interventions and study methods are described in detail elsewhere^24^. Once the centre was randomised, participants received the corresponding intervention training according to the assigned arm. A two-hour training workshop was conducted for both interventions before the trial commenced, followed by monthly internet-based training capsules tailored to each intervention. These capsules utilised clinical cases, medical literature, and reminders. Both intervention groups received both training programs.

The usual care group followed standard practice procedures. Training for enhanced communication skills emphasized information gathering on patient concerns, symptom exchange, disease understanding, antibiotics, and antimicrobial resistance. It included agreement on a management plan, safety measures, and ensuring patient comprehension. Clinicians were given interactive informative booklets to use during consultations, highlighting key points and offering them to patients. These patient booklets were developed based on findings from qualitative studies in LRTI patients before the trial^25,26^. Training on CRP rapid test usage included practical guidance on integrating test results during consultations in a two-hour session by the study team. Clinicians learned how to target testing in cases of clinical uncertainty, with an emphasis on ruling out serious infection for values below 20 mg/l. Further details are provided in the Supplementary Material.

### Procedures

Healthcare professionals were asked to recruit sequential eligible adults within regular consultation hours, starting on November 19, 2019. During the index consultation the participating professionals reported the duration of the illness, the severity of cough and other symptoms (rated 0, not problematic, 6, as bad as could be), clinical examination data, presence of addictions and comorbidities, quality of life data, initial treatment prescribed, diagnosis, whether CRP was tested or not, and whether the booklet was used or not. Apart from the point of care CRP tests, clinicians decided investigations and treatment according to their usual practice. Follow-up information about symptoms was reported by patients in self-completed diaries that were followed for two weeks. The diary also included several questions about secondary outcomes. Information on quality of life was collected by the healthcare professional at the days 15 and 45 in the case report form REDCap.

### Statistical analysis

Our study required 2940 patients with LRTI infection to detect a reduction in antibiotic prescribing from 60% to 45% (power 80%, α 0.05, follow-up 90%) when adjusted for clustering at a practice level (intracluster coefficient 0.07) as mentioned in the protocol^24^.

Continuous variables were described as means and standard deviations and categorical variables were expressed as percentages and frequencies. The study of the distribution of homogeneity of the patients according to baseline characteristics among the four trial arms was performed using the chi-square test or Fisher’s exact test for categorical variables, analysis of variance test for continuous variables, and Kruskal–Wallis test for discrete variables or non-normal continuous variables. Analyses were performed by intention to treat. A logistic regression model was used to assess antibiotic consumption within the first six weeks. The results were expressed in terms of odds ratio (OR) and the usual care arm was defined as the reference group. In addition, these ORs were adjusted for gender, age, smoking status, comorbidities of the patients, and the clustering effect using multilevel logistic regression. Specifically, the estimation was performed with the use of the generalised estimation method specifying a logit link function, binomial distribution, and exchangeable structure correlation. In relation to the EQ-5D-5L questionnaire score and visual analogue scale score, the differences between the 15 day and basal values across the four arms were analysed using a linear model. The parameters were estimated using the generalised estimation method specifying an identity link function, normal distribution, and exchangeable structure correlation. The results were expressed in terms of means. Analysis was performed using R software for Windows version 4.2.2 (R project for statistical computing; Vienna, Austria).

### Reporting summary

Further information on research design is available in the Nature Research Reporting Summary linked to this article.

### Supplementary information

Supplementary material

Reporting Summary

## Data Availability

The datasets generated and/or analysed during the current study are available in the SCIENTIA repository, http://scientiasalut.gencat.cat.
