# Supplementary material for: Effects of C-reactive protein rapid testing and communication skills training on antibiotic prescribing for acute cough. A cluster factorial randomised controlled trial
Source: NPJ Prim Care Respir Med. 2024 May 9;34:9. doi: 10.1038/s41533-024-00368-9 (PMC11081949; doi:10.1038/s41533-024-00368-9)
Supplement: Supplementary file 1 — Supplementary material [file 41533_2024_368_MOESM1_ESM.pdf]

SUPPLEMENTARY INFORMATION

Supplementary methods

Secondary outcomes

Secondary outcomes were antibiotic prescribing at the index consultation, duration of moderate to severe symptoms, number of reconsultations and complications, clinical recovery, number of days of sick leave and patient satisfaction and enablement. A new variable on antibiotic appropriateness was created on the basis of whether an antibiotic was given or not based on local clinical guidelines [1], considering that antibiotics are warranted for patients with pneumonia, bronchiectasis and for acute exacerbations of COPD when accompanied by purulent sputum and withheld in episodes of acute bronchitis and COPD exacerbations with non-purulent sputum. In addition, the efficiency of the interventions was evaluated separately.

Full description of the interventions

The interventions and study methods are described in detail elsewhere [reference 15, main manuscript]. An on-the-spot two-hour training workshop took place for both interventions before the inception of the trial. This training was followed by monthly internet-based short training capsules, tailored to either of the two interventions measured, with the use of clinical cases, medical literature, and reminders. Professionals assigned to both interventions received the two training programmes. The usual care group assessed and managed patients according to the normal practice procedures.

Training in enhanced communication skills were focused on the gathering of information on patients' concerns and expectations, exchange of information on symptoms, natural disease course and treatments, knowledge on the role of antibiotics and antimicrobial resistance, agreement of a management plan, security net, and checking of the understanding of the main points raised during the consultation. Clinicians were also provided with interactive informative booklets to use during consultations, emphasizing the most important parts with patients and with the possibility of giving them at the end of the consultation. Booklets included information on the natural history of LRTIs, the mean time of symptoms, use of antibiotics and their role in antibiotic resistance, self-help measures, and when to re-consult. These patient booklets were designed considering the results of two previous qualitative studies performed in patients with LRTIs before the trial started [references 16,17, main manuscript], and healthcare professionals were asked to emphasize the most important parts with patients and the booklets were given to the patients at the end of the consultation if required.

Training on the use of a CRP rapid test consisted of clear guidance on how to use the test results within the consultation during a two-hour practice-based training session delivered by the study team and they were trained on how to target testing, in cases of clinical uncertainty, such as in patients with high temperature, dyspnoea, abnormal auscultation, and impairment of vital signs, as well as in cases in which physicians perceived a patient demand for antibiotics. The benefit in ruling out serious infection with values lower than 20 mg/l was emphasised. The clinicians were given devices to test (Affinion 2, Abbott Diagnostics, United States), according to the manufacturer's instructions. A result can be available in less than three minutes, using a drop of blood obtained by finger prick. A four-week run-in period enabled familiarisation with the devices before patient recruitment to the trial commenced.

Supplementary results: Secondary outcomes

The prescribing rate at the index consultation was also significantly lower for patients in the communication skills training group compared to those allocated to usual care (25.5% vs. 50%). After adjusting for clustering age, sex, smoking status, and comorbidities, the effect continued being statistically significant (aOR 0.36 [95% CI 0.14 to 0.92]). Compared to those assigned to usual care, the prescribing rates at the index consultation were slightly lower, albeit not statistically significant, in the groups assigned to CRP rapid testing and both interventions combined when compared to the patients visiting professionals in the control group, with an aOR of 0.49 (95% CI 0.19 to 1.23) and 0.51 (95% CI 0.18 to 1.43), respectively (table 2, main manuscript). Delayed antibiotic prescribing was performed in seven cases (3% of all the contacts), all of whom were in the combined strategy group. General practitioners prescribed a wide range of antibiotics during the study, but especially β-lactams (J01C), with amoxicillin and amoxicillin-clavulanate being the most common antibiotics in 30 (36.6%) and 26 cases (31.7%), respectively. The rest of the cases corresponded to levofloxacin, azithromycin, ciprofloxacin, and clarithromycin. Compared to the usual care group, antibiotic appropriateness was slightly better in the communication skill enhancement group, with an aOR of 2.56 (95% CI 0.80 to 8.25), followed by the combination strategy group (1.65; CI 95% 0.54 to 5.05) and CRP group (1.40; 95% CI 0.36 to 5.42). Among healthcare professionals using the CRP test, antibiotic appropriateness was higher with extreme results, as the seven patients with CRP > 100 mg/l were prescribed antibiotics whereas antibiotic prescribing was withheld in 86.4% of the cases with low results (<20 mg/l).

The interventions had no discernible effect on recovery, resulting in comparable median daily symptom scores for all four groups of patients. Recovery was slightly better in the group of the

double intervention (see Supplementary Table S3). The interventions showed no statistically significant differences in reconsultations. Overall, satisfaction with the index consultation was high, with no statistically significant differences among treatment groups. The score for the patient enablement index was similar for all groups (see Supplementary Table S4).

References

1. Fernández Urrusuno R. Grupo de Trabajo de la Guía. Guía de Terapéutica Antimicrobiana del Área Aljarafe, 3rd ed. Sevilla. Distrito Sanitario Aljarafe-Sevilla Norte y Hospital de Dios de Aljarafe, 2018. Available at: http://www.juntadeandalucia.es/servicioandaluzdesalud/guiaterapeuticaaljarafe/guiaTerapeuticaAljarafe/

Supplementary table 1. Characteristics of the primary care centres participating in the trial.

Primary care centre	Arm assigned	Population covering in inhabitants	Socio-demographics, SDI, 2015*	Baseline antibiotic DID in 2018	Total number of GPs and nurses in the centre	Total number of participants in the trial	GPs partici-pating	Nurses partici-pating
Casernes	Communication skill enhancement	21,189	34.88	8.02	32	15	9	6
Sant Andreu	Communication skill enhancement	28,089	34.88	8.33	40	8	3	5
Chafarinas	Communication skill enhancement	18,895	69.98	9.55	22	11	7	4
Roger	Communication skill enhancement	18,473	38.3	6.77	25	13	5	8
Montnegre	Communication skill enhancement	24,638	14.83	7.76	28	6	4	2
Raval Nord	CRP rapid testing	20,528	54.92	7.83	28	7	5	2
Sant Rafael	CRP rapid testing	30,390	42.48	9.24	35	18	12	6
La Mina	CRP rapid testing	16,072	86.07	8.21	22	11	7	4
Pare Claret	CRP rapid testing	26,378	22.09	6.64	32	23	11	12
Adrià	CRP rapid testing	67,537	2.22	4.04	51	14	13	1
Casc Antic	Communication skills + CRP rapid testing	24,623	40.71	8.49	26	16	10	6
La Sagrera	Communication skills + CRP rapid testing	35,472	36.72	7.62	43	12	6	6
Carles Riba	Communication skills + CRP rapid testing	12,887	65.17	8.69	25	6	3	3
Passeig St Joan	Communication skills + CRP rapid testing	28,208	17.71	7.95	41	13	10	3
Sanllehy	Communication skills + CRP rapid testing	21,760	33.7	7.65	27	8	3	5
Guineueta	Usual care	25,775	51.75	7.99	35	11	9	2

Poble Sec	Usual care	21,847	45.84	7.6	29	10	7	3
Montcada	Usual care	33,432	54.6	9.1	34	11	11	0
Rio de Janeiro	Usual care	26,982	51.1	6.03	40	6	3	3
Numància	Usual care	24,110	25.05	5.94	29	12	11	1

CRP=C Reactive Protein; DID=Daily defined doses of systemic antibiotics (J01 group) per 1,000 Inhabitants per Day; GP=General Practitioner; SDI=Socio-Demographic Index; based on the 2015 socioeconomic index issued by the Catalan government (100: lowest socioeconomic status; 0: highest socioeconomic status). Based on: https://observatorisalut.gencat.cat/web/.content/minisite/observatorisalut/observatori_desigualtats/dades_obertes/Fitxers_crisi/Dades_indicador_socioeconomic_components_csv.csv

Supplementary table 2. Baseline symptoms and signs of the patients participating in the trial.

	All (n=233)	Communication skills training (n=56)	CRP training (n=48)	CRP + communication skill training (n=99)	Usual care (n=30)
Symptoms, median (IQR)					
Cough	4.0 (3.0-5.0)	4.0 (3.8-5.3)	5.0 (4.0-5.0)	3.0 (3.0-5.0)	4.0 (3.0-5.0)
Phlegm	4.0 (3.0-5.0)	3.5 (3.0-5.0)	4.0 (2.8-5.3)	3.0 (2.0-4.0)	4.0 (3.0-4.0)
Breathlessness	2.0 (0.0-4.0)	3.0 (0.0-5.5)	2.0 (0.0-4.3)	2.0 (0.0-3.0)	4.0 (1.8-4.3)
Wheezing	1.0 (0.0-5.0)	3.0 (0.0-6.3)	1.5 (0.0-5.3)	0.0 (0.0-2.0)	2.5 (0.0-4.3)
Chest pain†	0.0 (0.0-3.0)	1.0 (0.0-5.0)	3.0 (0.0-4.3)	0.0 (0.0-1.0)	1.0 (0.0-3.0)
Febrile sensation	1.0 (0.0-3.0)	2.5 (0.0-5.5)	3.0 (0.0-4.3)	0.0 (0.0-3.0)	0.0 (0.0-3.0)
General malaise	3.0 (2.0-5.0)	3.0 (1.8-5.3)	4.0 (2.0-5.0)	3.0 (2.0-5.0)	3.0 (2.0-4.3)
Difficulty in doing daily life activities†	3.0 (1.0-4.0)	3.5 (2.8-6.3)	4.0 (1.0-5.3)	1.0 (0.0-4.0)	2.0 (0.0-3.3)
Signs and tests, n (%)					
Sputum colour:					
- No sputum	38 (16.7)	8 (14.3)	8 (17.0)	18 (18.8)	4 (14.3)
- Transparent	65 (28.6)	22 (39.3)	8 (17.0)	27 (28.1)	8 (28.6)
- Purulent	124 (54.6)	26 (46.4)	31 (56.0)	51 (53.1)	16 (57.1)
Normal auscultation	80 (34.3)	25 (44.6)	13 (27.1)	34 (34.3)	8 (26.7)
Wheezing*	79 (33.9)	27 (48.2)	18 (37.5)	24 (24.2)	10 (33.3)
Ronchi†	88 (37.8)	8 (14.3)	26 (54.2)	37 (37.4)	17 (56.7)
Crackles*	28 (12.0)	1 (1.8)	4 (8.3)	21 (21.2)	2 (6.7)
Pulse oximetry	97.1 (1.7)	97.7 (1.2)	97.3 (1.6)	96.7 (1.9)	96.8 (1.6)
Abnormal X-chest ray	2 (25.0)	0 (0.0)	1 (33.3)	1 (33.3)	0 (0.0)

CRP=C Reactive Protein; IQR=Interquartile Range.

*p<0.05; †p<0.01

Supplementary table 3. Clinical recovery and complications at day 15.

	All (n=186)	Communication skills training (n=43)	CRP training (n=38)	CRP + communication skill training (n=79)	Usual care (n=26)
The cough is not or is only a minor problem*	142 (78.5)	28 (68.3)	26 (68.4)	66 (84.6)	22 (91.7)
The general malaise is not or is only a very little problem*	152 (85.9)	34 (85.0)	26 (74.3)	73 (93.6)	19 (79.2)
The daily activity has been recovered†	160 (91.4)	39 (95.0)	29 (82.9)	75 (97.4)	18 (78.3)
The patient has been on sick leave	33 (18.0)	4 (9.5)	8 (21.1)	16 (20.5)	5 (20.0)
Reconsultations during this period to:					
- Primary care	23 (12.4)	5 (11.6)	8 (21.1)	8 (10.1)	2 (7.7)
- Emergency department	8 (4.3)	2 (4.7)	3 (7.9)	3 (3.8)	0 (0.0)

CRP=C Reactive Protein.

*$p<0.05$; †$p<0.01$

Supplementary table 4. Satisfaction with the baseline visit and enablement.

	All (n=102)	Communication skills training (n=24)	CRP training (n=20)	CRP + communication skill training (n=38)	Usual care (n=20)
I would like to receive an antibiotic					
Totally agree	8 (8.0)	2 (8.3)	2 (10.0)	2 (5.26)	2 (11.1)
Agree	14 (14.0)	2 (8.3)	2 (10.0)	6 (15.8)	4 (22.2)
Indifferent	36 (36.0)	11 (45.8)	3 (15.0)	16 (42.1)	6 (33.3)
Disagree	18 (18.0)	3 (12.5)	6 (30.0)	3 (7.9)	6 (33.3)
Totally disagree	12 (12.0)	5 (20.8)	3 (15.0)	4 (10.5)	0 (0.0)
No answer	12 (12.0)	1 (4.17)	4 (20.0)	7 (18.4)	0 (0.0)
I consider that I need an antibiotic					
Totally agree	12 (11.9)	4 (16.7)	1 (5.0)	4 (10.5)	3 (15.8)
Agree	21 (20.8)	2 (8.3)	4 (20.0)	7 (18.4)	8 (42.1)
Indifferent	40 (39.6)	12 (50.0)	4 (20.0)	17 (44.7)	7 (36.8)
Disagree	9 (8.9)	1 (4.2)	6 (30.0)	1 (2.6)	1 (5.3)
Totally disagree	7 (6.9)	4 (16.7)	1 (5.0)	2 (5.3)	0 (0.0)
No answer	12 (11.9)	1 (4.17)	4 (20.0)	7 (18.4)	0 (0.0)
I come to obtain an antibiotic					
Totally agree					
Agree	8 (8.0)	2 (8.3)	1 (5.0)	3 (7.9)	2 (11.1)
Indifferent	7 (7.0)	2 (8.3)	1 (5.0)	1 (2.6)	3 (16.7)
Disagree	24 (24.0)	8 (33.3)	1 (5.0)	14 (36.8)	1 (5.6)
Totally disagree	24 (24.0)	5 (20.8)	6 (30.0)	6 (15.8)	7 (38.9)
No answer	24 (24.0)	6 (25.0)	7 (35.0)	7 (18.4)	4 (22.2)
	13 (13.0)	1 (4.17)	4 (20.0)	7 (18.4)	1 (5.6)
I have received a complete information about my disease and treatment					
Totally agree	48 (47.1)	11 (45.8)	8 (40.0)	17 (44.7)	12 (60.0)

Agree	32 (31.4)	8 (33.3)	6 (30.0)	12 (31.6)	6 (30.0)
Indifferent	1 (1.0)	1 (4.2)	0 (0.0)	0 (0.0)	0 (0.0)
Disagree	1 (1.0)	0 (0.0)	0 (0.0)	0 (0.0)	1 (5.0)
Totally disagree	0 (0.0)	0 (0.0)	0 (0.0)	0 (0.0)	0 (0.0)
No answer	20 (19.6)	4 (16.7)	6 (30.0)	9 (23.7)	1 (5.0)
After the visit I am more aware of my health problem much better					
Totally agree	41 (40.2)	9 (37.5)	9 (45.0)	16 (42.1)	7 (35.0)
Agree	35 (34.3)	8 (33.3)	5 (25.0)	13 (34.2)	9 (45.0)
Indifferent	6 (5.9)	3 (12.5)	0 (0.0)	0 (0.00)	3 (15.0)
Disagree	0 (0.0)	0 (0.0)	0 (0.00)	0 (0.0)	0 (0.0)
Totally disagree	0 (0.0)	0 (0.0)	0 (0.0)	0 (0.0)	0 (0.0)
No answer	20 (19.6)	4 (16.7)	6 (30.0)	9 (23.7)	1 (5.0)
I will follow the advice given because I think it is accurate					
Totally agree	47 (46.1)	8 (33.3)	8 (40.0)	20 (52.6)	11 (55.0)
Agree	34 (33.3)	12 (50.0)	6 (30.0)	8 (21.1)	8 (40.0)
Indifferent	0 (0.0)	0 (0.0)	0 (0.0)	0 (0.0)	0 (0.0)
Disagree	0 (0.0)	0 (0.0)	0 (0.0)	0 (0.0)	0 (0.0)
Totally disagree	1 (1.0)	0 (0.0)	0 (0.0)	1 (2.6)	0 (0.0)
No answer	20 (19.6)	4 (16.7)	6 (30.0)	9 (23.7)	1 (5.0)
Some aspects of the visit could have been better					
Totally agree	10 (9.8)	3 (12.5)	1 (5.0)	6 (15.8)	0 (0.0)
Agree	18 (17.6)	6 (25.0)	6 (30.0)	4 (10.5)	2 (10.0)
Indifferent	17 (16.7)	2 (8.3)	3 (15.0)	8 (21.1)	4 (20.0)
Disagree	22 (21.6)	4 (16.7)	4 (20.0)	6 (15.8)	8 (40.0)
Totally disagree	15 (14.7)	5 (20.8)	0 (0.0)	5 (13.2)	5 (25.0)
No answer	20 (19.6)	4 (16.7)	6 (30.0)	9 (23.7)	1 (5.0)
I am satisfied with the visit					
Totally agree	53 (52.0)	10 (41.7)	8 (40.0)	22 (57.9)	13 (65.0)
Agree	27 (26.5)	9 (37.5)	6 (30.0)	7 (18.4)	5 (25.0)

Indifferent	2 (2.0)	1 (4.2)	0 (0.0)	0 (0.0)	1 (5.0)
Disagree	0 (0.0)	0 (0.0)	0 (0.0)	0 (0.0)	0 (0.0)
Totally disagree	0 (0.0)	0 (0.0)	0 (0.0)	0 (0.0)	0 (0.0)
No answer	20 (19.6)	4 (16.7)	6 (30.0)	9 (23.7)	1 (5.0)

CRP=C Reactive Protein.
